# A systematic review of ultrasonography-guided transcutaneous fine needle aspiration cytology in the diagnosis of laryngeal malignancy

**DOI:** 10.1308/rcsann.2024.0095

**Published:** 2024-11-15

**Authors:** A Ahmed, D Yang, M Eastwood, T Saunders, SF Ahsan

**Affiliations:** ^1^ Northern Care Alliance NHS Foundation Trust, UK; ^2^ Shrewsbury and Telford Hospital NHS Trust, UK

**Keywords:** laryngeal cancer, ultrasonography guided, fine needle aspiration

## Abstract

**Introduction:**

Direct laryngoscopy and biopsy is the gold standard for obtaining a tissue diagnosis in patients with suspected laryngeal cancer. In patients with advanced disease or other medical comorbidities, this may come with significant anaesthetic risks, including tracheostomy. Ultrasonography-guided biopsy has been widely used in the diagnosis of malignancy involving cervical lymph nodes but it is not commonly employed in the diagnosis of laryngeal tumours. A systematic review was undertaken to assess the literature looking at whether ultrasonography-guided transcutaneous fine needle aspiration cytology (FNAC) is an adequate method in diagnosing laryngeal malignancy.

**Methods:**

Two independent researchers conducted a systematic review of the literature using the MEDLINE^®^ and Cochrane Library databases in accordance with the PRISMA (Preferred Reporting Items for Systematic reviews and Meta-Analyses) guidelines.

**Results:**

A total of 568 studies were identified from the search, of which 3 met the inclusion criteria, resulting in 162 patient episodes. The pooled accuracy of transcutaneous FNAC in acquiring a sample adequate for histological diagnosis was 74.9%. Data on complications were limited, with a few cases of mild haemoptysis being recorded.

**Conclusions:**

Transcutaneous FNAC can be considered a safe and quick method for establishing a histological diagnosis of laryngeal lesions, particularly in patients who may be severely comorbid, and it could therefore could reduce the risks of general anaesthesia and tracheostomy prior to commencing definitive treatment.

## Introduction

Fine needle aspiration cytology (FNAC) is an established method of obtaining histological diagnosis in malignancy, particularly in cervical lymphadenopathy. It involves the examination of cells obtained by passing a needle through a target lesion. Lesions affecting the larynx can be difficult to access and are therefore typically assessed using direct laryngoscopy, which requires general anaesthesia. This allows facilitation of biopsy for tissue diagnosis as well as enabling operative planning and potential debulking of obstructive disease. Lesions may also be biopsied using transnasal pharyngo-oesophagoscopy although this technology is not yet available in every department.

However, severely comorbid patients may not be suitable for biopsy under general anaesthesia. Moreover, patients who have extensive laryngeal masses may require protective tracheostomy or emergency tracheostomy to secure the airway in cases of post-intubation or procedural oedema. This may have implications for morbidity and outcome, particularly in the context of radiotherapy.^1,2^

The aim of this study was to review the literature to assess whether ultrasonography-guided transcutaneous FNAC is an adequate and safe method of obtaining histological diagnosis in laryngeal tumours without the need for general anaesthesia.

## Methods

The study protocol was registered on the PROSPERO prospective database of systematic reviews (CRD42020216045). The review has been reported in accordance with the 2020 PRISMA (Preferred Reporting Items for Systematic reviews and Meta-Analyses) guidelines.^3^

### Study inclusion criteria

Clinical studies of patients undergoing ultrasonography-guided transcutaneous FNAC for diagnosis of suspected laryngopharyngeal cancer were included. Studies of any experimental or observational design in humans were included. Animal studies and human studies not reporting adequacy or accuracy rate for FNAC, or where the abstract or full text were unavailable in the English language were excluded.

### Search strategy

**Figure 1:**
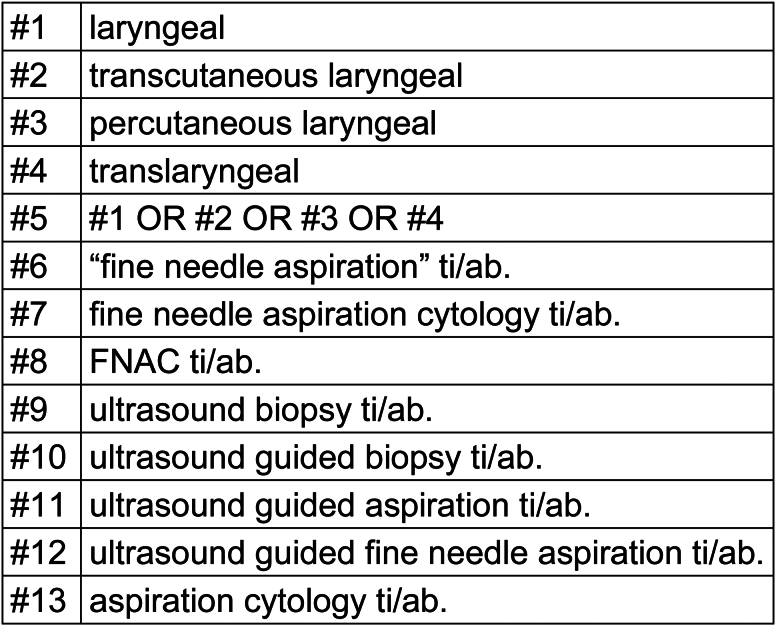
Search terms

Two reviewers (ME and DY) independently performed the literature searches and screened abstracts. The following databases were searched: MEDLINE^®^, PubMed^®^, Embase^®^, Web of Science™, Cochrane Library and ClinicalTrials.gov (via Cochrane). The search terms used are listed in Figure 1. No limit was placed on language or year of publication.

### Selection process

Two reviewers (DY and ME) independently screened the title and abstract of all records identified from the database searches. Studies describing ultrasonography-guided transcutaneous FNAC in patients with suspected laryngopharyngeal cancer were assessed against the inclusion and exclusion criteria, with any disagreement resolved by discussion with a third reviewer (TS). Studies without an accessible abstract or full text after the title/abstract screening were followed up by attempting to contact the study authors. If they remained unavailable, the study was excluded. Studies using other methods of imaging guidance or tissue sampling were also excluded, as were any not reporting sample adequacy. Studies in which transcutaneous FNAC outcomes were grouped with those of other methods were followed up by contacting the study authors. If data remained undistinguishable, the study was excluded. Studies presenting overlapping or duplicate populations were limited to the largest study sharing data where it was not possible to disambiguate them.

Potentially relevant studies identified from the initial searches and abstract screening then underwent full-text screening by the two independent reviewers prior to data extraction. Conflicts on selection were resolved by discussion between the reviewers.

### Data extraction

Data were extracted by one reviewer (DY) and subsequently checked by a second reviewer (ME). Extracted data were collected in an Excel^®^ spreadsheet (Microsoft, Redmond, WA, US).

### Risk of bias quality scoring

Two reviewers independently assessed the risk of bias using the Brazzelli risk of bias checklist for non-randomised studies.^4^ Studies were also evaluated according to the Oxford Centre for Evidence Based Medicine (OCEBM) grading system.^5^ Discrepancies between the reviewers were resolved by discussion.

## Results

### Study selection

**Figure 2:**
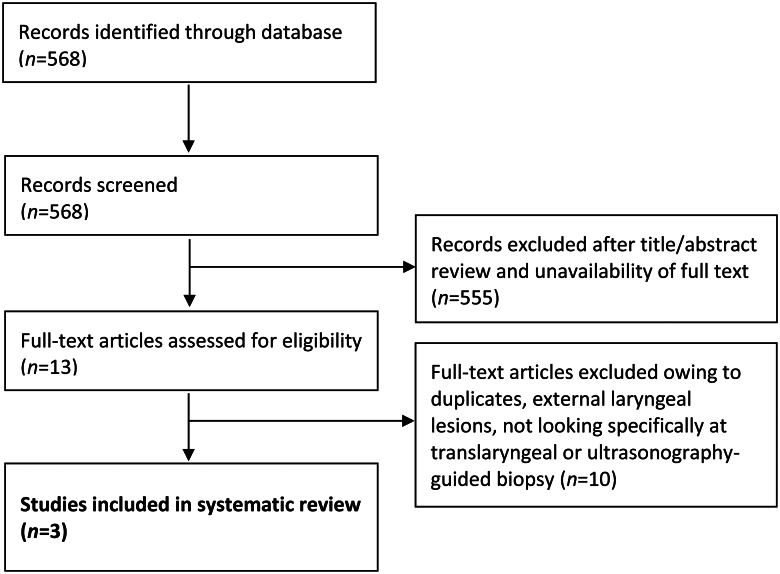
Flowchart of study selection process

A total of 568 studies were identified from the literature search, of which 3 met the inclusion criteria.^6–8^ The process of identifying and screening records is displayed in Figure 2.

### Study characteristics

The three studies included in this review involved a total of 215 patients. Two of the studies were cohort analyses from India: a prospective study of 24 patients and a retrospective study of 189 patients. Also included was a retrospective case series of two patients in the US. The studies were published between 2013 and 2020. The study characteristics including FNAC technique and OCEBM level of evidence^5^ are summarised in Table 1.

**Table 1:** Study characteristics

Study	Country	Study type	Number of patients	Average age (range)	FNAC technique	OCEBM grade^5^
Parasuraman, 2020^6^	India	Prospective cohort	24	56.5 years(40–73 years)	25G needle, ultrasonography-guided	3
Mukundapai, 2018^7^	India	Retrospective cohort	189	54.5 years(20–89 years)	21–23G needle,ultrasonography/CT-guided	3
Lopchinsky, 2013^8^	US	Retrospective case series	2	66 yearsx(62–70 years)	21G needle, ultrasonography-guided	4
CT = computed tomography; FNAC = fine needle aspiration cytology; OCEBM = Oxford Centre for Evidence-Based Medicine						

### Results of individual studies

The results of each individual study are summarised in Table 2. Parasuraman *et al* demonstrated a diagnostic accuracy rate of FNAC gaining a cytological diagnosis of 87.5%.^6^ Of the 24 patients included, 21 samples showed squamous cell carcinoma (SCC), 2 samples demonstrated atypical cells and 1 sample contained inadequate material for analysis. The three latter patients proceeded to have direct laryngoscopy and biopsy, and were then confirmed to also have SCC. FNAC was reported to be tolerated well by all patients with no complications.

**Table 2:** Study results

Study	Adequate sample for analysis	Cytomorphological diagnosis obtained	Overall rate of diagnosis	Diagnoses obtained	Further biopsy results
Parasuraman, 2020^6^	23/24 (95.6%)	21/23 (91.3%)	21/24 (87.5%)	• 21 SCC • 2 atypical cells • 1 inadequate material for cytology	• 2 atypical cells and 1 inadequate material:all confirmed SCC on laryngoscopy and biopsy
Mukundapai, 2018^7^	146/189 (77.2%)	138/146 (94.5%)	138/189 (73.0%)	• 122 SCC • 1 PA • 1 ACC • 6 non-neoplastic • 8 poorly differentiated carcinoma • 8 descriptive reports	• 8 poorly differentiated carcinoma:2 confirmed SCC on biopsy, 3 patients died,3 lost to follow-up • 8 descriptive reports: 2 confirmed SCC,6 lost to follow-up • 43 inadequate material: 13 confirmed SCC,30 lost to follow-up
Lopchinsky, 2013^8^	2/2 (100%)	2/2 (100%)	2/2 (100%)	• 2 SCC	N/A
ACC = adenoid cystic carcinoma; PA = pleomorphic adenoma; SCC = squamous cell carcinoma					

Mukundapai *et al* investigated the largest cohort, with 189 patients; 146 patients provided satisfactory aspirate material while the remaining 43 patients were unable to provide adequate samples for cytology.^7^ Of the satisfactory samples, 132 were labelled neoplastic, 6 samples were benign and 8 cases were given descriptive labels such as necrosis or mucinous material. Among the samples identified as neoplastic, eight patients were labelled as having poorly differentiated carcinoma, possibly high-grade SCC. The descriptive reports and poorly differentiated samples tended to be investigated further to confirm diagnosis. However, only four cases were biopsied and confirmed as SCC, three patients died prior to biopsy and the remaining patients were lost to follow-up. Mukundapai *et al* were able to ascertain a cytomorphological diagnosis in 138 patients, giving a diagnostic accuracy rate of 73.0%. They reported a small number of cases of mild haemoptysis but otherwise no complications from the procedure.

The case series reported by Lopchinsky *et al* included two patients who underwent ultrasonography-guided FNAC and who were both successfully diagnosed with SCC, giving a 100% adequacy rate for the case series.^8^ No complications were mentioned by the authors.

### Synthesis of results

Pooling of the results of the 215 patients from the included studies shows that 171 patients produced adequate samples for cytological analysis. Of these, 161 patient samples were able to lead to a diagnosis. This gives an overall adequacy rate of 74.9%.

### Risk of bias

All included studies were graded at level 3 or 4 of the OCEBM levels of evidence.^5^ The risk of bias assessment is presented in Table 3. Certain studies had a high risk of bias due to retrospective data collection and lack of sufficient follow-up.

**Table 3:** Risk of bias assessment using checklist by Brazzelli *et al*^4^

Criteria	Parasuraman, 2020^6^	Mukundapai, 2018^7^	Lopchinsky, 2013^8^
Study type	Cohort	Cohort	Case series
1. Were participants a representative sample selected from a relevant patient population (e.g. randomly selected from those seeking treatment despite age, duration of disease, primary or secondary disease and severity of disease)?	No	No	No
2. Were the inclusion/exclusion criteria of participants clearly described?	Yes	Yes	Not available
3. Were participants entering the study at a similar point in their disease progression (i.e. severity of disease)?	Yes	Unclear	No
4. Was selection of patients consecutive?	Unclear	Unclear	Unclear
5. Was data collection undertaken prospectively?	Yes	No	No
6. Were the groups comparable on demographic characteristics and clinical features?	Not available	Not available	Not available
7. Was the intervention (and comparison) clearly defined?	Yes	Yes	Yes
8. Was the intervention undertaken by someone experienced at performing the procedure?	Yes	Unclear	Yes
9. Were the staff, place and facilities where the patients were treated appropriate for performing the procedure (e.g. access to back-up facilities in hospital or special clinic)?	Yes	Unclear	Yes
10. Were any of the important outcomes considered (i.e. on clinical effectiveness, cost effectiveness or learning curves)?	Yes	Yes	Yes
11. Were objective (valid and reliable) outcome measures used, including satisfaction scale?	Yes	No	No
12. Was the assessment of main outcomes blind?	Not available	Not available	Not available
13. Was follow-up long enough (≥1 year) to detect important effects on outcomes of interest?	No	Yes	No
14. Was information provided on non-respondents/dropouts?	Not available	Yes	Not available
15. Were the characteristics of withdrawals/dropouts similar to those that completed the study and therefore unlikely to cause bias?	Not available	Yes	Not available
16. Was length of follow-up similar between comparison groups?	Not available	Not available	Not available
17. Were the important prognostic factors identified (e.g. age, duration of disease, disease severity)?	Yes	No	No
18. Were the analyses adjusted for confounding factors?	Not available	Not available	Not available

## Discussion

This systematic review and narrative synthesis reports on the utility of ultrasonography-guided FNAC as a method for diagnosing laryngeal malignancy. To our knowledge, this is the first systematic review of this topic.

### Interpretation of results

The studies included in our review all report positive results for the use of FNAC as a method of reaching a histological diagnosis for laryngeal malignancy, citing the safety and speed of the procedure. This suggests that FNAC could offer an alternative to direct laryngoscopy and biopsy. Although two studies were retrospective and only one was prospective, it was deemed reasonable to pool the results across all three studies as the study population and intervention in each study appeared to be similar.

In all studies, FNAC was performed successfully in most patients, with minimal complications. Parasuraman *et al* found it to be successful in almost all patients, demonstrating FNAC to be a viable option for individuals who are not fit for direct laryngoscopy under general anaesthesia.^6^

Mukundapai *et al* had a greater rate of sample inadequacy, which was attributed to several factors, such as size and site of lesion, and uncooperative patients.^7^ The reason for this difference in sample adequacy may be due to the individual study designs and patient selection criteria. Mukundapai *et al* used a retrospective design looking at all patients who were investigated by FNAC in a larger tertiary centre. These patients were not chosen based on selection criteria and were instead investigated by FNAC as a primary investigation of their tumour. Lopchinsky *et al* reported on a case series demonstrating successful use of FNAC in patients who were not candidates for operative laryngoscopy and biopsy.^8^

In the context of other evidence, FNAC also carries proven use in the diagnosis of other head and neck conditions such as thyroid pathology. One retrospective study from 2023 conducted by Osseis *et al* looked at the diagnostic accuracy of FNAC in thyroid tumours, using histopathology as a reference standard.^9^ It was found that FNAC had an overall accuracy rate of 75.89%, demonstrating moderate diagnostic accuracy.

### Limitations

The limitations of our systematic review should be considered when interpreting its results. The study by Mukundapai *et al* is limited by its retrospective design^7^ and that by Lopchinsky *et al* is limited as it is a case series.^8^ Mukundapai *et al* included all patients investigated with FNAC and did not include any specific selection criteria to isolate certain target populations. Conversely, as the study by Parasuraman *et al* had a prospective design, this meant specific selection criteria were set for patients who were likely to produce better results (i.e. mainly patients with advanced laryngeal and hypopharyngeal mass lesions [T3 and T4 lesions]).^6^ They also excluded patients with calcified thyroid cartilage, vascular lesions and lesions not detected by ultrasonography. This could have skewed their results in favour of higher diagnostic accuracy.

Neither Parasuraman *et al* nor Lopchinsky *et al* provided information on patient follow-up or long-term outcomes,^6,7^ Moreover, FNAC was not compared directly with laryngoscopy and biopsy in any of the three studies in our review.^6–8^

Despite these limitations, all three studies included representative samples of the target population, gave sufficient information on how patients were selected for inclusion and had clearly defined outcome measures (adequacy and diagnostic accuracy of FNAC).^6–8^ Patients were selected consecutively within similar age ranges and all FNAC was performed by adequately trained staff in appropriate environments.

### Implications for future practice

Across all three studies, if only including the patients for whom adequate samples for analysis were obtained, the average diagnostic accuracy for FNAC increases from 74.9% to 94.2%, meaning that when an adequate sample is produced, FNAC offers a high diagnostic yield. Parasuraman *et al* suggest immediately assessing samples for adequacy prior to analysis as a method to ensure adequate samples.^6^ In fact, guidelines from the National Institute for Health and Care Excellence recommend the presence of a cytopathologist at the time of aspiration for this specific purpose, and many hospitals have implemented ‘one stop’ head and neck clinics to facilitate this in other head and neck malignancies.^10^ Consequently, a ‘one stop’ clinic in which diagnostic FNAC is used for laryngeal malignancy could be an excellent way to achieve quick and safe diagnoses while avoiding general anaesthesia.

## Conclusions

Our analysis of three studies investigating the use of transcutaneous FNAC as a diagnostic tool for laryngeal lesions suggests that it may be a quick, safe and accurate method for establishing histological diagnosis, especially when adequate aspirate samples are achieved. It invites discussion around a ‘one stop’ head and neck clinic in future practice, which may avoid anaesthetic risks in comorbid patients. However, further prospective studies with larger numbers and adequate follow-up are required to compare the adequacy of diagnostic FNAC with laryngoscopy and biopsy for laryngeal malignancy, in particular looking at long-term outcomes, diagnostic accuracy, time to treatment and risk of perioperative complications.
